# Design and Characterisation of a Read-Out System for Wireless Monitoring of a Novel Implantable Sensor for Abdominal Aortic Aneurysm Monitoring

**DOI:** 10.3390/s24103195

**Published:** 2024-05-17

**Authors:** Nuno P. Silva, Adnan Elahi, Eoghan Dunne, Martin O’Halloran, Bilal Amin

**Affiliations:** 1 Translational Medical Device Lab, University of Galway, H91 TK33 Galway, Ireland; adnan.elahi@universityofgalway.ie (A.E.); eoghandonncha.dunne@universityofgalway.ie (E.D.); martin.ohalloran@universityofgalway.ie (M.O.); bilal.amin@universityofgalway.ie (B.A.); 2 Electrical and Electronic Engineering, University of Galway, H91 TK33 Galway, Ireland; 3 School of Medicine, University of Galway, H91 TK33 Galway, Ireland

**Keywords:** abdominal aortic aneurysm, EVAR surveillance, magnetic inductive coupling, wireless implantable medical sensor, Z-shaped inductor

## Abstract

Abdominal aortic aneurysm (AAA) is a dilation of the aorta artery larger than its normal diameter (>3 cm). Endovascular aneurysm repair (EVAR) is a minimally invasive treatment option that involves the placement of a graft in the aneurysmal portion of the aorta artery. This treatment requires multiple follow-ups with medical imaging, which is expensive, time-consuming, and resource-demanding for healthcare systems. An alternative solution is the use of wireless implantable sensors (WIMSs) to monitor the growth of the aneurysm. A WIMS capable of monitoring aneurysm size longitudinally could serve as an alternative monitoring approach for post-EVAR patients. This study has developed and characterised a three-coil inductive read-out system to detect variations in the resonance frequency of the novel Z-shaped WIMS implanted within the AAA sac. Specifically, the spacing between the transmitter and the repeater inductors was optimised to maximise the detection of the sensor by the transmitter inductor. Moreover, an experimental evaluation was also performed for different orientations of the transmitter coil with reference to the WIMS. Finally, the FDA-approved material nitinol was used to develop the WIMS, the transmitter, and repeater inductors as a proof of concept for further studies. The findings of the characterisation from the air medium suggest that the read-out system can detect the WIMS up to 5 cm, regardless of the orientation of the Z-shape WIMS, with the detection range increasing as the orientation approaches 0°. This study provides sufficient evidence that the proposed WIMS and the read-out system can be used for AAA expansion over time.

## 1. Introduction

Abdominal aortic aneurysm (AAA) is characterised by the abnormal dilation of the abdominal segment of the aorta, exceeding 1.5 times its standard diameter (more than 3 cm) [1,2]. Clinically, an AAA greater than 5.5 cm is considered at high risk of rupture [3,4]. The Society of Vascular Surgery indicates an annual rupture rate of 30–50% for aneurysms larger than 7 cm [5]. Nevertheless, there are documented cases in the literature where AAA size exceeds 10 cm, referred to as giant AAA [6]. Annually, around 3 million individuals receive a diagnosis of AAA. This condition is listed among the top 10 causes of mortality in the USA and among the top 15 in several European nations [2,7].

Current clinical practices widely utilise endovascular aneurysm repair (EVAR) as a viable treatment option. In cases where a patient is not suitable for EVAR, open surgery is necessary to repair the affected artery [8]. EVAR is a minimally invasive procedure that involves placing a graft in the aneurysmal area using a catheter to redirect blood flow through the graft [2,8]. EVAR offers a survival advantage of the patient’s life in the early stages compared to open surgery. However, EVAR often leads to subsequent interventions or repairs in the long term [9] due to complications related to the graft or the occurrence of endoleaks (blood backflow into the aneurysm following EVAR). Therefore, ongoing surveillance post-EVAR is essential in order to monitor graft function and detect endoleaks, as they are linked to aneurysm expansion and an increased risk of aneurysm rupture [9].

Follow-up procedures commonly involve utilising imaging techniques like computed tomography (CT) angiography, magnetic resonance imaging (MRI), or duplex ultrasound (DUS) [10]. Contrast agents are typically required for MRI and CT scans, which pose risks of nephrotoxicity and radiation exposure, respectively [11]. In addition, these imaging methods are costly, time-consuming, and resource-intensive for healthcare systems [12]. While MRI and CT scans are widely used, DUS is increasingly being recognised for its ability to detect endoleaks and measure the diameter of the aneurysm during post-EVAR follow-ups [13]. DUS offers advantages such as reduced radiation exposure and lower costs. Nevertheless, studies indicate that CTA remains the preferred imaging modality due to CTA having a higher sensitivity than DUS, which stands at 50% [13,14].

Hence, health systems require a secure, cost-effective, and efficient solution for monitoring the growth of aneurysms post-EVAR. One potential approach is to employ wireless monitoring through implantable sensors inserted into the aneurysm for continuous monitoring [15,16]. Over the last twenty years, implantable intra-pressure sensing devices have been explored as a monitoring technology post-EVAR to track AAA progression [15,16]. The primary goal behind designing these implantable pressure sensors was to establish a correlation between arterial pressure and aneurysm sac pressure variances, aiming to investigate if these pressure changes could be associated with a specific type of endoleak [15]. Despite successful pressure measurements, no significant correlation was found between the presence of endoleaks, aneurysm growth, or their absence [15,16]. Consequently, implantable pressure sensors were not integrated into clinical practice [17]. Among healthcare practitioners, aneurysm size remains the most crucial factor in predicting aneurysm rupture [18]. An implantable sensor capable of monitoring the size of the cross-section of the aneurysm could serve as an alternative monitoring approach for post-EVAR patients. Various types of wireless implantable medical sensors (WIMSs) have been proposed to cater to diverse clinical requirements for optical, neurological, and cardiovascular conditions [19,20]. Moreover, studies indicate that stent grafts can be adapted to function as intelligent sensors tailored to specific applications, such as detecting blood clots or blood flow [21,22,23,24]. Many of these devices utilise wireless inductive coupling for sensor power supply and data recording of essential physiological parameters. The development of such implantable sensors offers real-time monitoring capabilities, enabling medical professionals to remotely monitor patients from any location and without restricting their activities [19,20,25]. This remote monitoring feature allows healthcare teams to oversee patients without the necessity of them visiting medical facilities.

Wireless inductive coupling systems function based on the concept that energy can be transferred between an external inductor and an implantable inductor (sensor) when they are brought into proximity [26]. Research conducted over the past few decades has focused on optimising and maximising these energy transfer systems and their associated features [19,20,25,27]. These investigated features pertain to the electrical properties of the inductors within the system, including self-inductance, resistance, parasitic capacitance, and quality factor [27]. The effectiveness of the coupling between the external inductor and the implantable inductor depends on these characteristics.

Various read-out systems have been suggested in the literature for wirelessly powering WIMSs in a range of applications, such as supporting pacemakers and implantable sensors [28,29]. Some of these systems are capable of both transmitting and receiving data from the implanted device [30]. Different types of read-out systems, specifically transmitter inductors, have been proposed to enhance the coupling efficiency between an external read-out inductor and the WIMS. The two-coil inductive link is the most commonly used configuration for powering or retrieving data from WIMS [28,31]. This two-coil setup achieves optimal power transfer efficiency only within the near-field region (around 2 cm) [31]. For longer distances, multi-coil inductive links utilising three- or four-coil configurations are typically preferred for their high efficiency in communication with the implantable sensor [32]. Despite the selection of the inductive link configuration, there is insufficient information regarding the spacing between the transmitter and receiver inductors that would maximise coupling with the implantable sensor. Moreover, the alignment between the transmitter–repeater unit and the sensor remains unclear.

To detect the WIMS for AAA size detection, this study addresses the development and characterisation of a read-out system. The read-out system is designed to be placed external to the body to detect the Z-shaped sensor, which is intended to be deployed in the aneurysmal sac. The multi-coil inductive read-out system was composed of a transmitter and a repeater inductor. The optimal spacing between the transmitter and repeater inductors was characterised to maximise the detection of the Z-shaped inductor. Moreover, different alignment angles between the Z-shaped inductor and external read-out system were analysed.

To develop this sensor, the methodology used is presented in Section 2, including the design of the read-out system and its characterisation. Section 3 presents the results and discussion of this study. Finally, Section 4 presents the conclusion of this study and proposes directions for future work.

## 2. Methodology

A read-out system for the detection of WIMS is composed of several components which allow the coupling and, consequently, the reading of the WIMS. The main component that establishes the coupling between the read-out system and the WIMS is the inductor [27]. The inductor is usually created using geometries such as circular loops or spiral planar coils [27]. These geometries have been well studied and analysed in the literature. However, the optimisation of the read-out inductors for reading data from a specific type of WIMS is performed by adapting the geometries of these inductors. This optimised geometry aims to increase the efficiency of the communication link between the external read-out inductor and the implantable sensor. In addition to the common 2-coil link methodology, studies have proposed the use of repeater inductors to increase the efficiency of coupling between external and implantable inductors [32]. Repeater inductors are used in the read-out system to increase the power of the signal received from the WIMS, as observed in other studies [30,32].

**Figure 1:**
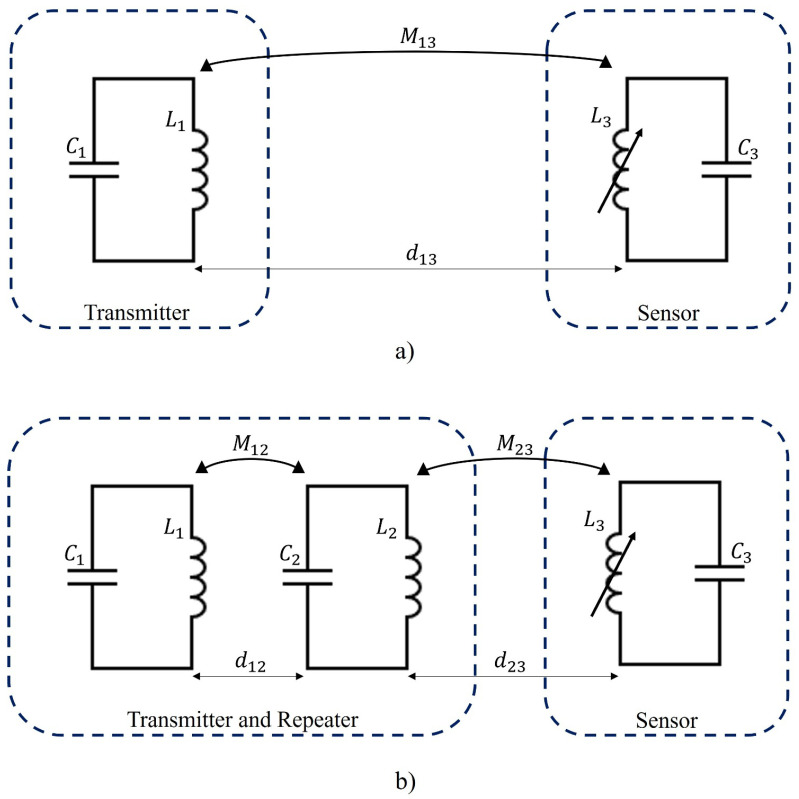
A schematic circuitry diagram of the (**a**) 2-coil inductive link and (**b**) 3-coil inductive link. $L_{1}$, $L_{2}$, and $L_{3}$ correspond to the transmitter, repeater, and receiver (sensor) inductors, respectively, presented in the circuitry, while $C_{1}$, $C_{2}$, and $C_{3}$ correspond to the capacitors of the transmitter, receiver, and sensor circuits. The 2-coil inductive link methodology is characterised by the presence of just two inductors which act as transmitter and receiver. On the other hand, the 3-coil inductive link is characterised by the presence of an extra inductor that acts as a repeater inductor between the transmitter and receiver inductors.

This study has considered both 2- and 3-coil inductive link methodologies to detect the Z-shaped WIMS proposed in [33]. The 2-coil inductive link does not have the presence of the repeater inductor, as shown in Figure 1a, while the 3-coil system consists of two external inductors and an implantable inductor, as shown in Figure 1b. From Figure 1b, it can observed that the two external inductors (i.e., the transmitter, $L_{1}$, and repeater, $L_{2}$, inductors), are part of the read-out system, while the other inductor of the 3-coil inductive link is the WIMS, $L_{3}$, (i.e., Z-shaped inductor).

This 3-coil inductive link technique enables the read-out system to be optimised through the geometry of each inductor as well as the spacing between the transmitter and repeater inductors [32]. In this topology, it is anticipated that the increase in mutual coupling between the transmitter and repeater inductors, $M_{12}$, will result in an increase in the mutual coupling between the read-out system and the implantable sensor, $M_{23}$ [32]. The increase in the mutual coupling between inductors is extremely relevant in the optimisation process because this change increases the efficiency of the read-out system detecting the sensor.

Therefore, this section presents the design and analysis of the read-out system in order to detect the sensor at different distances. The following subsections describe the inductors presented in the read-out system. Furthermore, an experimental evaluation of the detection of the Z-shaped sensor is also described considering different orientations.

### 2.1. Proposed Geometries for the Read-Out Inductor System

**Figure 2:**
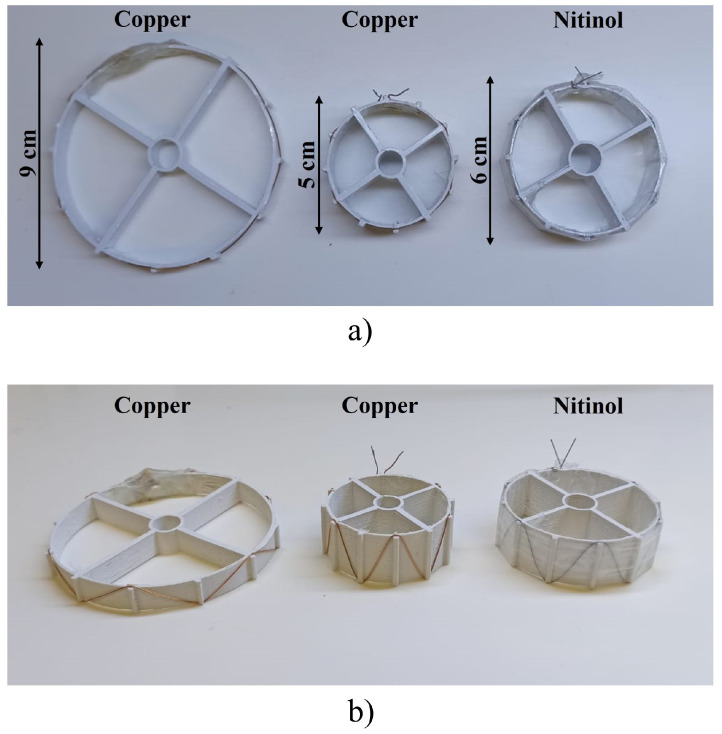
The design of the Z-shaped inductors from the (**a**) top and (**b**) isometric perspectives. The Z-shaped inductors are constituted of a sequential ring of six struts. Two Z-shaped inductors were manufactured in copper, with diameters of 9 and 5 cm. Another Z-shaped inductor was manufactured using a biocompatible material, nitinol, with a diameter of 6 cm. The Z-shaped inductors were manufactured using a 3D-printed PLA structure to keep the inductor intact.

The geometries of the transmitter and repeater inductors, $L_{1}$ and $L_{2}$, respectively, were based on standard geometries. These standard geometry configurations allow the inductors to be characterised for desired inductance values using well-studied analytical methods. In this case, the transmitter is a circular loop inductor, whereas the repeater inductor is a spiral planar inductor. The selection of these two configurations was based on prior research. These two configurations in the read-out system enable us to power an implantable sensor [30,32]. The read-out inductors are shown in Figure 2.

The geometry of each inductor is depicted following their respective characteristics, as outlined in Table 1. Both inductors were created using copper with a thickness of 1 mm and with the geometric characteristics defined in Table 1. The inductance of the inductors was measured using the Keysight E4990A impedance analyser, with the Keysight 16047E test fixture. The error between the measured and theoretical inductances was less than 10%, as reported in Table 1. After the inductance was determined at the target frequency of 13.56 MHz, the choice of a frequency of 13.56 MHz was made based on its widespread adoption in implantable medical sensors, as evidenced by prior studies [28,32,34,35,36]. This frequency is recognized and sanctioned as a safe operational range for medical devices, as it does not pose risks of tissue damage stemming from either radiation exposure or thermal effects [37]. Furthermore, existing research indicates that electromagnetic waves generated by the inductor penetrate organic tissues more effectively at frequencies below 100 MHz [20,38], reinforcing the suitability of the chosen frequency for ensuring adequate tissue penetration and minimising potential adverse effects. Consequently, the incorporation of capacitors is imperative to ensure that inductors resonate at the same resonance frequency. The capacitors were chosen to comply with the same resonant frequency. A capacitor of 440 pF was soldered to the terminals of the transmitter inductor, $L_{1}$, while a capacitor of 47 pF was soldered to the terminals of the repeater inductor, $L_{2}$. From the equation of resonant frequency ($f=1/2\pi \sqrt{LC}$, where *L* and *C* are the inductance and capacitance, respectively), the resonant frequency values of the inductors were established to be 13.3 and 13.6 MHz, respectively, for the transmitter and receiver inductors.

To preserve the shape of the transmitter and receiver inductors, 3D-printed polylactic acid (PLA) frames were fabricated (Figure 2) to maintain the shape of the inductor and facilitate measurement, without impact on the coupling. The design of each inductor was drawn with computer-aided design (CAD) software (Fusion 360, Autodesk, CA, USA). Afterwards, the drawing was imported to the BCN3D Stratos 1.5.3 (BCN3D, Barcelona, Spain) software. Finally, the frames were printed in PLA with a nozzle size of 0.4 mm using a 3D printer, Epsilon W50 (BCN3D, Barcelona, Spain).

### 2.2. Z-Shaped Inductors for Wireless Linkage Characterisation

**Figure 3:**
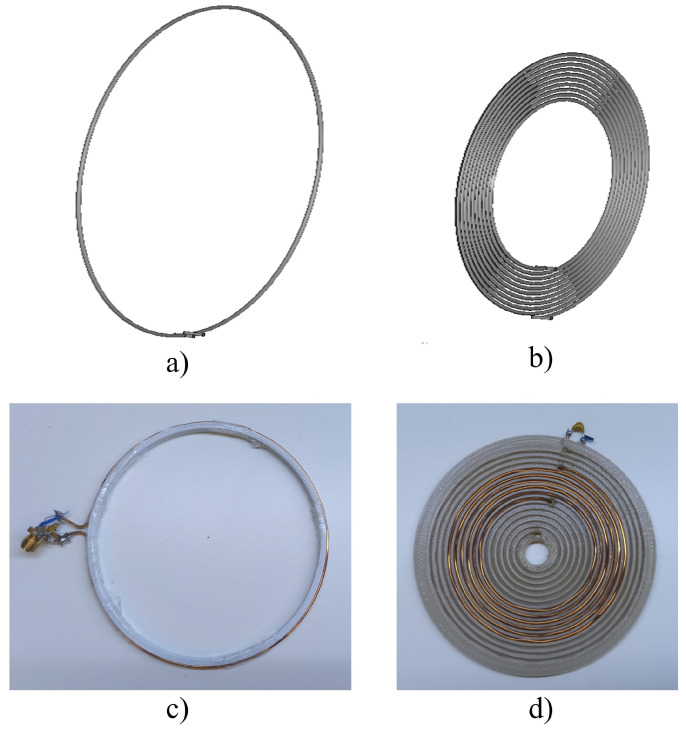
The schematic design of the (**a**) transmitter ($L_{1}$) and (**b**) repeater ($L_{2}$) inductors of the read-out system, for the detection of the Z-shaped inductor, in an isometric view. The design of the (**c**) transmitter ($L_{1}$) and (**d**) repeater ($L_{2}$) (top view) inductors of the read-out system. Capacitors were soldered at the terminals of the inductors, and an SMA male connector was soldered at the terminal of the transmitter inductor. Both inductors were created using a 3D-printed PLA framework to sustain the configuration of the inductors.

The Z-shaped inductor was proposed in our previous research work [33]. This implantable Z-shaped inductor was designed following a sequential ring stent-based shape. This shape allows the inductor to expand according to the growth of AAA. The Z-shaped inductor changes its inductance as the radius and the height change. This inductance–geometry relationship is demonstrated in the previous work conducted by Silva et al. [33]. This change in inductance will adjust the resonance frequency of the sensor, allowing us to translate it into a size value. Two different scenarios were considered when selecting the sizes of the aneurysms. The first scenario represented an aneurysm at the threshold for EVAR, with a diameter of 5 cm. In addition, a larger aneurysm measuring 9 cm in diameter was chosen as the second size. These two sizes of aneurysms represent two different scenarios that can manifest in these patients. Additionally, a Z-shaped inductor composed of a six-strut sequential ring was created for testing purposes, as shown in Figure 3. The geometric characteristics of both Z-shaped sensors are shown in Table 2.

Similar to frames designed for the Z-shaped inductor, frames were created for the read-out system, as shown in Figure 3. The two structures were defined with radii of 2.5 and 4.5 cm, and the height was calculated according to the equation proposed for the height by Silva et al. [33], as shown in Table 2. The Z-shaped sensor was then created using the exact same methodology and materials described by Silva et al. [33].

Besides the copper-based sensors, another sensor was developed utilising nitinol for biocompatibility, which was a nickel and titanium alloy [39]. In this instance, the sensor was tested with the biocompatible material. This biocompatible metal exhibits two closely related and unique properties: shape memory effect and superelasticity [39]. Shape memory is the ability of the material to undergo deformation at one temperature, stay in its deformed shape when the external force is removed, and then recover its original undeformed shape by heating it above its “transformation temperature” [39,40]. Superelasticity is the ability of the metal to undergo large deformations and immediately return to its undeformed shape upon the removal of the external load. This material is widely used in medical devices such as stents [40,41]. The characteristics of the sensor are the same as those developed for the copper-based Z-shaped inductor and can be observed in Table 2.

The three Z-shaped inductors created were initially evaluated using a Keysight E4990A impedance analyser (Keysight Technologies, CA, USA) and the Keysight 16047E test fixture. The inductance of the inductors was determined to select the correct capacitors to be soldered at the terminals of the Z-shaped inductors. The selected capacitor was soldered in all the Z-shaped inductors to mimic the correct change in the resonant frequency due to the change in the geometry of the sensor with the expansion of the aneurysm. In this case, a 550 pF ceramic capacitor was soldered to the terminals of each Z-shaped inductor, obtaining a resonant frequency of 11.4 and 13.5 MHz for the large and medium Z-shaped inductors manufactured in copper, respectively, and a resonant frequency of 12.4 MHz for the Z-shaped inductor manufactured in nitinol.

### 2.3. The Experimental Evaluation of the Read-Out Inductor for the Detection of the Z-Shaped Inductor

The experimental evaluation of the read-out system in detecting the Z-shaped sensor was carried out by measuring the reflection coefficient parameter (|$S_{11}$|) of the transmitter inductor of the read-out system. To observe the detection of the sensor, the magnitude of the |$S_{11}$| signal provided by the transmitter inductor was tested by changing the distance from the repeater inductor. This variability aims to select the distance from these two inductors which optimises the detection of the Z-shaped sensor.

**Figure 4:**
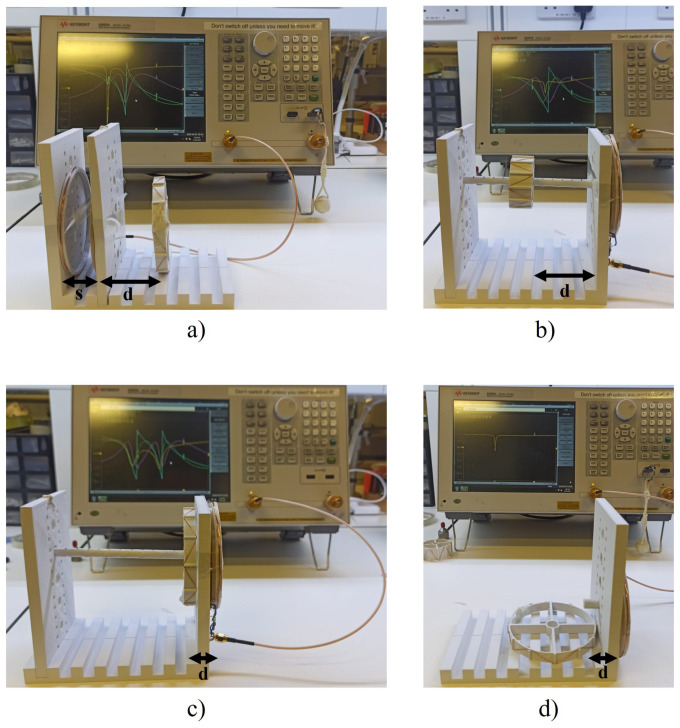
An illustration of the experimental evaluation of the reading system and the evaluated sensors, according to the spacing between the transmitter and repeater inductors (*s*) and the distance between the reading system and the sensor (*d*). The evaluation of the variability between the transmitter and repeater inductors is represented in (**a**). In (**b**,**c**), the evaluation of the medium and large sensors align at 0° with the reading system for distances between 1 and 10 cm, respectively, while (**d**) represents the same evaluation but with the sensor aligned at 90°.

To increase the mutual coupling between both the transmitter and repeater inductors, it is important to determine the best spacing between them. Thus, the spacing between the transmitter and the repeater was analysed to observe the point at which the mutual coupling was maximised, by measuring |$S_{11}$|. |$S_{11}$| was analysed considering a fixed distance of 5 cm between the repeater inductor and the sensor. The distance between the repeater inductor and the Z-shaped sensor was fixed to guarantee the exact same detection conditions from the read-out system. The only condition evaluated then was the spacing between the transmitter and the repeater inductor. Subsequently, the spacing between the transmitter and the repeater inductors was varied from 0 to 4 cm. These measurements were conducted in an air medium. The transmitter was connected to the Keysight E5063A Vector Network Analyser (VNA) (Keysight Technologies, Santa Rosa, CA, USA). Then, the spacing between the transmitter and receiver inductors was gradually increasing. The experimental evaluation is shown in Figure 4a. The mutual inductance was considered maximised when the detection of the sensor represented the maximum magnitude of |$S_{11}$|.

Furthermore, an auxiliary plastic assembly was developed to streamline the process of quantifying inter-inductor distances, as seen in Figure 4a. This auxiliary plastic assembly was created to minimise the possible movements of the read-out system or the sensor and to allow us to keep the orientation of the inductors stable during the experimental evaluation.

The distance between the read-out system and the sensor was studied within the air medium. When two inductors are aligned (horizontally aligned with an angle of 0° between them), the maximum power transfer efficiency occurs between them, resulting in the maximum interaction between inductors. A decrease in efficiency is observed when the inductors are misaligned. Furthermore, the likelihood of the angle approaching 90° between the read-out system and the sensor is extremely high. Anatomically, the sensor is placed within the aneurysm in the abdominal portion of the aorta. As the abdominal portion of the aorta is orientated vertically, this implies that the sensor will be oriented 90° relative to the external abdominal wall. This study considered two different angles between the read-out system and the sensor: 0° and 90°, as shown in Figure 4. The measurement of the s-parameters for sensor detection was carried out similarly to the procedure explained above. The transmitter inductor was connected to the VNA and the repeater inductor was placed right beside it. Subsequently, the sensor was positioned according to the intended angle alignment, and various distances ranging from 1 to 10 cm were studied. The experimental evaluation was carried out for the three Z-shaped inductors described in Section 2.2.

## 3. Results and Discussion

The experimental evaluation results are outlined in this section. Initially, the analysis of the variability in the spacing between the inductors within the read-out system is presented. Subsequently, the examination of the read-out system’s detection capabilities concerning the Z-shaped inductor is reported. In this study, the feasibility of detection of the implantable sensor was based upon the magnitude of the dip observed in the $S_{11}$ parameter at the designated target frequency of 13.56 MHz. The magnitude of the dips of the $S_{11}$ parameter directly correlates with the detection outcomes for the Z-shaped inductor by the read-out system, with lower magnitudes of the $S_{11}$ parameter’s dips indicating enhanced detection sensitivity and performance between the implantable sensor and the read-out system.

### 3.1. The Spacing Optimisation between the Read-Out System Inductors

**Figure 5:**
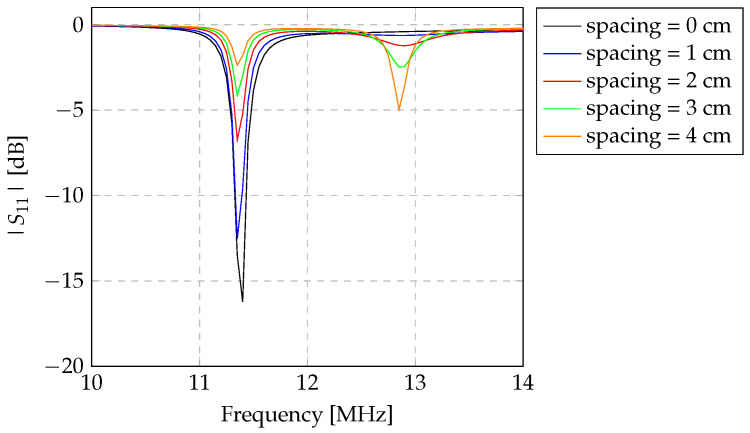
|$S_{11}$| [dB] of the variability between the transmitter and receiver inductors according to their spacing. The Z-shaped inductor was fixed at 5 cm from the repeater inductor. The resonance frequency of the used Z-shaped inductor is equal to 11.4 MHz. It is observed that the reflection from |$S_{11}$| [dB] is higher at that frequency, when transmitter and repeater inductors are at 0 mm distance between each other.

To maximise the detection of the Z-shaped inductor by the read-out system (composed of two inductors), the spacing between the inductors in the read-out system was evaluated. As described in Section 2.3, the |$S_{11}$| parameter from the transmitter inductor was evaluated at different distances from the repeater inductor, with the Z-shaped inductor fixed at a distance of 5 cm from the repeater inductor. The results related to the variability of the spacing between the transmitter inductor ($L_{1}$) and the repeater ($L_{2}$) are shown in Figure 5.

In Figure 5, the distinctions in |$S_{11}$| due to the alteration in the transmitter–repeater spacing are evident, while the Z-shaped inductor remains at constant separation from the repeater inductor. In this instance, the Z-shaped inductor was chosen to represent a resonance frequency different from the read-out system in order to understand the impact of the repeater inductor on the transmitter signal. The resonant frequency of the Z-shaped inductor with a diameter of 9 cm is 11.4 MHz, as identified in Table 2, while the read-out system has a resonant frequency of approximately 13.3 MHz, as identified in Table 1.

From the observation of Figure 5, it becomes apparent that the transmitter inductor detects an augmented reflected signal from the sensor inductor as the repeater is brought closer to the transmitter. Conversely, as the spacing between the inductors of the read-out system increases, the detection of the sensor diminishes, while the reflection of the signal due to the repeater inductor amplifies. This effect becomes very visible at the resonance frequency of the read-out system. According to the data derived from the measurements, heightened detection of the repeater inductor within the transmitter inductor occurs as the spacing between them increases. Consequently, it results in a reduction in sensor inductor detection. Moreover, it is important to emphasise that for distances greater than 0 cm between the inductors of the read-out system, the presence of the repeater inductor becomes evident. In this case, it is noticeable that this interaction occurs at a frequency of 12.8 MHz, which means that there is a shift in the resonant frequency of the read-out system by 0.5 MHz, i.e., of 3.8%. The shift in the resonant frequency during the coupling of the inductors occurs due to an increase in the coupling with the transmitter inductor.

These findings suggest that a 0 cm spacing between the inductors of the read-out system allows the repeater inductor to act as a signal amplifier, thereby optimising the detection of sensors by the transmitter inductor.

### 3.2. The Analysis of the Detection of the Z-Shaped Inductor by the Read-Out Inductors System

After finding the optimal spacing between the transmitter and the repeater inductors to maximise sensor detection, a new evaluation was performed to observe the detection of different Z-shaped inductors according to the distance from the read-out system. As explained in Section 2.3, the read-out system was evaluated with three different Z-shaped inductors at different distances and orientations. This experiment aims to understand the detection of Z-shaped inductors of different sizes, materials, and orientations.

#### 3.2.1. The Analysis of the Z-Shaped Inductor Orientation

**Figure 6:**
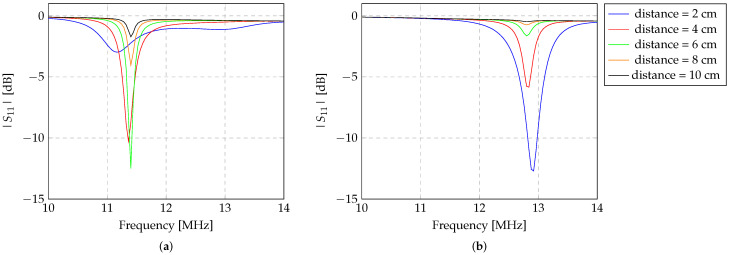
Experimental evaluation of the detection of the (**a**) large (diameter of 9 cm) and (**b**) medium (diameter of 5 cm) Z-shaped sensors. The evaluation was conducted in the air, with the sensor aligned with the transmitter and repeater inductors.

**Figure 7:**
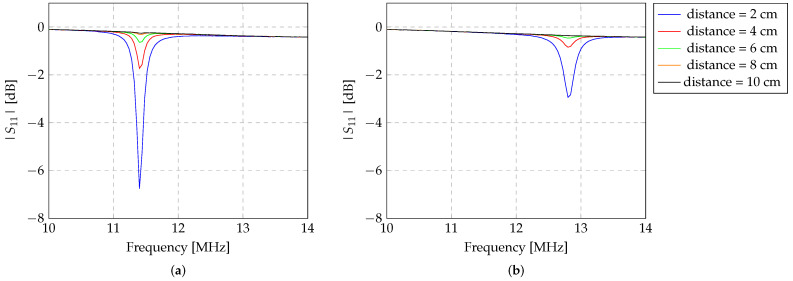
Experimental evaluation of the detection of the (**a**) large (diameter of 9 cm) and (**b**) medium (diameter of 5 cm) Z-shaped sensors. The evaluation was conducted in the air, with the sensor aligned with the transmitter and repeater inductors at an angle of 90°.

Figure 6 and Figure 7 show the experimental results of the evaluation of the read-out system for the detection of two Z-shaped copper inductors (sensors) of varying sizes: one large and one medium (as characterised in Table 2 and detailed in Section 2.2). The experimental evaluation of sensor detection was performed following the methodology elucidated in Section 2.3. The Z-shaped inductor was placed at distances ranging from 1 to 10 cm. |$S_{11}$| was measured on the transmitter inductor of the read-out system for varying distances, maintaining the angle at 0°. The experimental evaluations were conducted within an air medium, with the sensor aligned at both 0° and 90° angles relative to the read-out system, as depicted in Figure 4c and Figure 4d, respectively. The graphical representations in Figure 6 and Figure 7 show the results, accounting for the 0° and 90° angles between the Z-shaped inductor and the read-out system, respectively.

When the sensor is perfectly aligned with the reading system, as illustrated in Figure 6, it becomes apparent that for the larger Z-shaped inductor, detection remains viable up to a distance of at least 10 cm. However, a slight deviation in resonance frequency is noticeable for sensor detection, particularly at distances less than 4 cm. This frequency shift, concerning the resonance frequency, stands at 4.3% when the sensor is positioned 1 cm from the reading system, decreasing as the distance increases. This discrepancy is attributed to the proximity of the sensor to the reading system, engendering an interconnection between the three inductors. Notably, this frequency shift is not observed for the medium-sized sensor, nor when both sensors are aligned at a 90° angle, as is evident in Figure 7.

In the case of the medium-sized Z-shaped inductor, shown in Figure 6b and Figure 7b, detection is feasible only up to an 8 cm distance between the sensor and the reading system. This observation underscores that as sensors diminish in dimension, their detection necessarily occurs within closer proximity to the reading system. Furthermore, it is noteworthy from Figure 6b and Figure 7b that a significant drop in detection is evident when the sensor is aligned at a 90° angle with the reading system, a phenomenon observed previously for the larger sensor.

#### 3.2.2. Analysis Using a Two-Coil and Three-Coil Inductive Link

**Figure 8:**
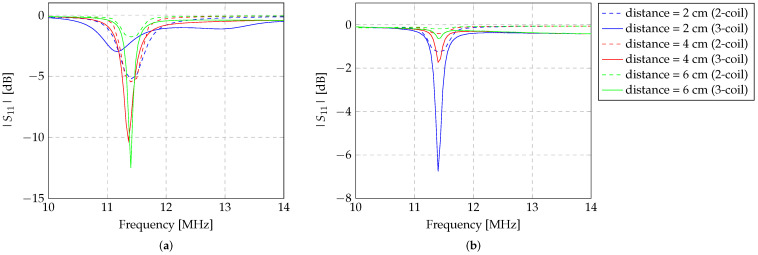
Experimental evaluation of the detection of the large (diameter of 9 cm) Z-shaped sensor by two different typologies for the inductive (2-coil and 3-coil inductive). The evaluation was conducted in the air, with the sensor (**a**) aligned (0° angle) with the two different typologies for the inductive, and (**b**) with the sensor orientated at 90° with the reader system.

Additionally, the read-out system was also evaluated considering only the transmitter inductor. In this case, a two-coil topology inductive link was experimentally evaluated at the same conditions as described previously for the three-coil topology. Figure 8 presents the graphical representation of the two-coil topology link, between the transmitter and the large Z-shaped inductor, for the orientations of 0° and 90° angles.

As observed in Figure 8, the three-coil topology link presents better results on the detection of the Z-shaped inductor than the two-coil topology. The presence of a repeater inductor increases the mutual coupling between the read-out system (composed of the transmitter and repeater inductors) and the Z-shaped inductor. This finding allows an increase in the magnitude signal detection of the Z-shaped inductor by the transmitter inductor of the read-out system.

#### 3.2.3. Analysis Using a Biocompatible Material

**Figure 9:**
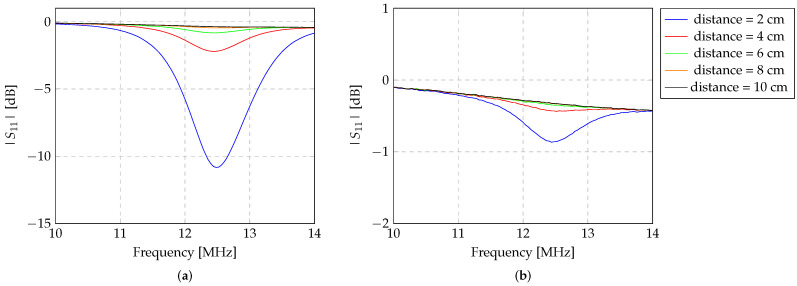
Experimental evaluation of the detection of a Z-shaped sensor created with nitinol with diameter of 6 cm. The evaluation was conducted in the air, with the sensor aligned with the transmitter and repeater inductors at (**a**) 0° and (**b**) 90°.

Figure 9 depicts the experimental assessment of detecting a sensor with a biocompatible material. This material is used in several medical devices for human implantation [40,41]. This material, nitinol, is particularly used in stents and other equipment due to the memory shape property of nitinol. This sensor, with its geometric characteristics outlined in Table 2, underwent experimental evaluation as well, similar to the previous experimental evaluation conducted with the manufactured copper Z-shaped inductors.

**Figure 10:**
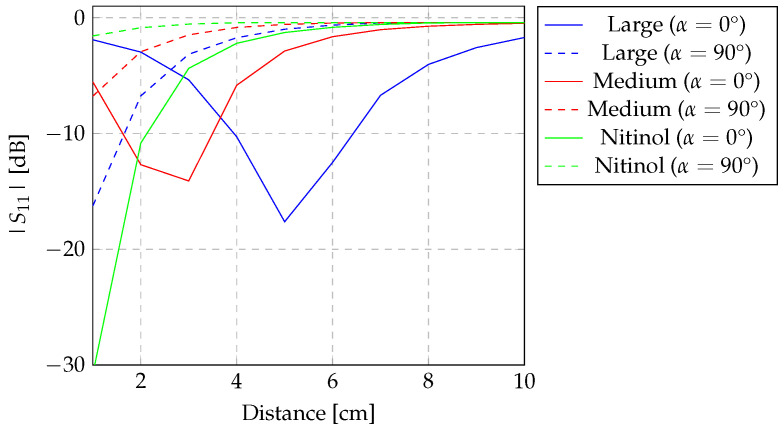
|$S_{11}$| [dB] of the sensor under test, accordingly with their distance to read-out system. The |$S_{11}$| [dB] exploited agree with the resonance frequency of each sensor, detected by the transmitter inductor.

As is evident from Figure 9, this type of material enables sensor detection, albeit in a much less pronounced manner compared to a copper sensor (Figure 6 and Figure 7). In both alignments with the reading system, the nitinol sensor allows detection up to approximately 5 cm, nearly equivalent to the diameter of the sensor. In Figure 10, the |$S_{11}$| values are depicted as a function of the distance between the sensor and the reading system. The figure represents the |$S_{11}$| values at the resonance frequency of each tested sensor, considering their alignment angles.

As observed in Figure 10, the detection of the sensor by the reading system follows a decreasing pattern as the distance between the sensor and the reading system increases. An exception is noticeable when the sensor is aligned at 90° with the reading system. In this case, it can be observed that the sensor is more easily detected when the sensor is at a distance approximately equal to its radius; however, the same observation is not verified in the case of the sensor with biocompatible material. Moreover, it is observed that the large sensor, representing large-sized aneurysms, is detected at all distances when aligned at 0° with the sensor. When aligned at 90°, the sensor becomes difficult to detect by the reading system at a distance of approximately 8 cm from the transmitter inductor. Despite the challenging detection of the sensor at a long distance when aligned at 90°, it is important to note that the literature does not predict coupling between inductors when they are aligned at this angle. Furthermore, it can be observed that for this angle, the detection distance decreases as the sensor size decreases.

Table 3 provides an overview of analogous studies employing diverse inductor configurations within the read-out system to interface with various designs of implantable sensors. The analysis of Table 3 reveals that the detection capabilities of the Z-shaped inductor by the proposed read-out system align with those of previous studies employing similar frequencies and a three-coil inductive link. Specifically, our system demonstrates the capacity to detect the sensor at distances of up to 50 mm, which represents an edge case of the maximum separation between the sensor and read-out system for the proposed application. Moreover, comparative assessments with studies utilising two-coil inductive links, corroborated in Figure 8 in our study, underscore the superior distance detection capabilities afforded by the proposed system. Furthermore, our read-out system exhibits the ability to detect the Z-shaped inductor even when oriented at a 90° angle to the read-out system. Crucially, for the specific application of AAA monitoring, wherein the detection of the implantable Z-shaped inductor is paramount, a requisite distance of approximately 2.1 cm is deemed necessary. This determination is based on considerations of the distance between the body surface (skin) and the abdominal segment of the aorta artery, utilising a body model representative of a “standard male body” measuring 1.77 m in height and weighing 72.4 kg, as established by Christ et al. [42]. Notably, the experimental results conducted in an air medium demonstrate that the Z-shaped inductor exhibits detectability at distances exceeding the aforementioned requirement. However, the imperative for further investigations to comprehensively analyse and characterise the proposed system in biological tissue environments is underscored, necessitating subsequent studies to investigate its efficacy in realistic ex vivo scenarios.

## 4. Conclusions

In this study, we introduce a read-out system based on the design principles of a three-coil inductive link system. The purpose of this system is to position the read-out system external to the body. Then, the read-out system aims to detect variations in the resonant frequency of a Z-shaped inductor implanted within the AAA sac. The effective coupling between the read-out system and the sensor results in the reading of the size of the aneurysm. This study aimed to investigate the optimal spacing between the transmitter and repeater inductors of the read-out system. In addition, the study also aimed to investigate the performance of the read-out system in the detection of the Z-shaped inductor at different angles. Also, the proposed read-out system underwent examination concerning the detection of both medium and large Z-shaped sensors comprised of copper, as well as a Z-shaped sensor crafted from a biocompatible material, nitinol.

Primarily, we present the results concerning the optimisation of the spacing between the transmitter and repeater inductors of the read-out system. These findings indicate that maximal detection is achieved when the transmitter and repeater inductors are positioned in close proximity, specifically at a distance of 0 cm. Moreover, a comparison between the two-coil and three-coil topology inductive links was conducted using a large Z-shaped inductor for detection. It was observed that the three-coil inductive link enhances the detection of the Z-shaped inductor compared to the alternative typology.

The optimal spacing between the transmitter and repeater inductors was found to be close to each other. Then, the orientation experiment assessed the detection of the Z-shaped inductor in an air medium when aligned with the read-out system at 0° and 90°. The results indicate that the Z-shaped inductor can be readily detected when horizontally aligned with the read-out system. However, the detected signal weakens as the orientation of the inductor shifts to 90°, becoming more easily detectable up to a distance of 5 cm from the read-out system.

The findings from air medium experiments regarding the detection of the Z-shaped inductor suggest that the read-out system is capable of detecting the sensor up to 5 cm regardless of the orientation of the Z-shaped inductor, with detection range increasing as the orientation approaches 0°. While the proposed read-out system was demonstrated to successfully detect the Z-shaped implantable inductor in air medium, it is important to note that the coupling efficiency between the read-out system and the implantable inductor may reduce under realistic tissue conditions. Although the selected operating frequency minimises the EM losses in the presence of biological tissues, it is imperative to validate the detection capabilities of the proposed system in a realistic biological environment.

This wireless implantable medical sensing technology can be compared with other similar technologies that have been investigated to act as surveillance methods for AAA post-EVAR procedure. Despite the established efficacy of implantable pressure-sensing devices in measuring sac pressures following endovascular aneurysm repair, these devices have not been shown to correlate pressure measurements with changes in aneurysmal sac growth or endoleak development. The accurate monitoring of aneurysmal sac growth is a crucial aspect of post-EVAR surveillance for abdominal aortic aneurysms. However, the current reliance on complex and expensive imaging procedures can result in prolonged waiting times, which may compromise patient safety and treatment outcomes. Therefore, there exists a pressing clinical need for innovative monitoring technologies that can address these limitations and provide timely and accurate assessments of AAA growth. The proposed sensing mechanism and read-out system address this clinical need by providing a novel solution to monitoring aneurysm growth post-EVAR. This innovative technology has the potential to improve patient outcomes and enhance the success of endovascular treatments for AAA by enabling the timely detection of changes in aneurysmal sac size, shape, and press.

## Figures and Tables

**Table 1:** The transmitter and receiver inductors’ specifications, where $L_{1}$ and $L_{2}$ are the transmitter and repeater inductors, respectively.

Parameter	$L_{1}$	$L_{2}$
Outer diameter [mm]	100	75
Inner diameter [mm]	-	55
Number of turns	-	5
Width [mm]	-	1
Spacing between turns [mm]	-	1
Wire radius [mm]	0.5	0.5
Type of wire	Copper	Copper
Inductance measured [nH]	320	3070
Inductance calculated [nH]	294	2950
Error [%]	9	5
Capacitor [pF]	440	47
Frequency [MHz]	13.4	13.2

**Table 2:** Z-shaped inductors’ specifications.

Parameter	$L_{3}$		
Inductance [nH]	354	251	299
Diameter [mm]	90	50	60
Height [mm]	10.4	22.0	20.3
Number of struts	6	6	6
Capacitor [pF]	550	550	550
Wire radius [mm]	0.250	0.250	0.235
Type of wire	Copper	Copper	Nitinol
Frequency [MHz]	11.4	13.5	12.4

The $L_{3}$ entries correspond to a large, medium, and nitinol Z-shaped inductors.

**Table 3:** Comparison with conventional works.

Parameter	Inductive Link	Frequency [MHz]	Distance [mm]	Ref.
Cardiac pacemaker	3-coil	13.56	50	[32]
Cardiac pacemaker	2-coil	13.56	40	[43]
Implantable sensor	3-coil	65	30	[25]
Implantable sensor	2-coil	0.3	10	[35]
Implantable sensor	2-coil	13.56	10	[35]
Temperature implantable sensor	3-coil	0.18	50	[44]
Z-shaped implantable sensor	3-coil	13.56	>50	-

## Data Availability

Data are contained within the article.
